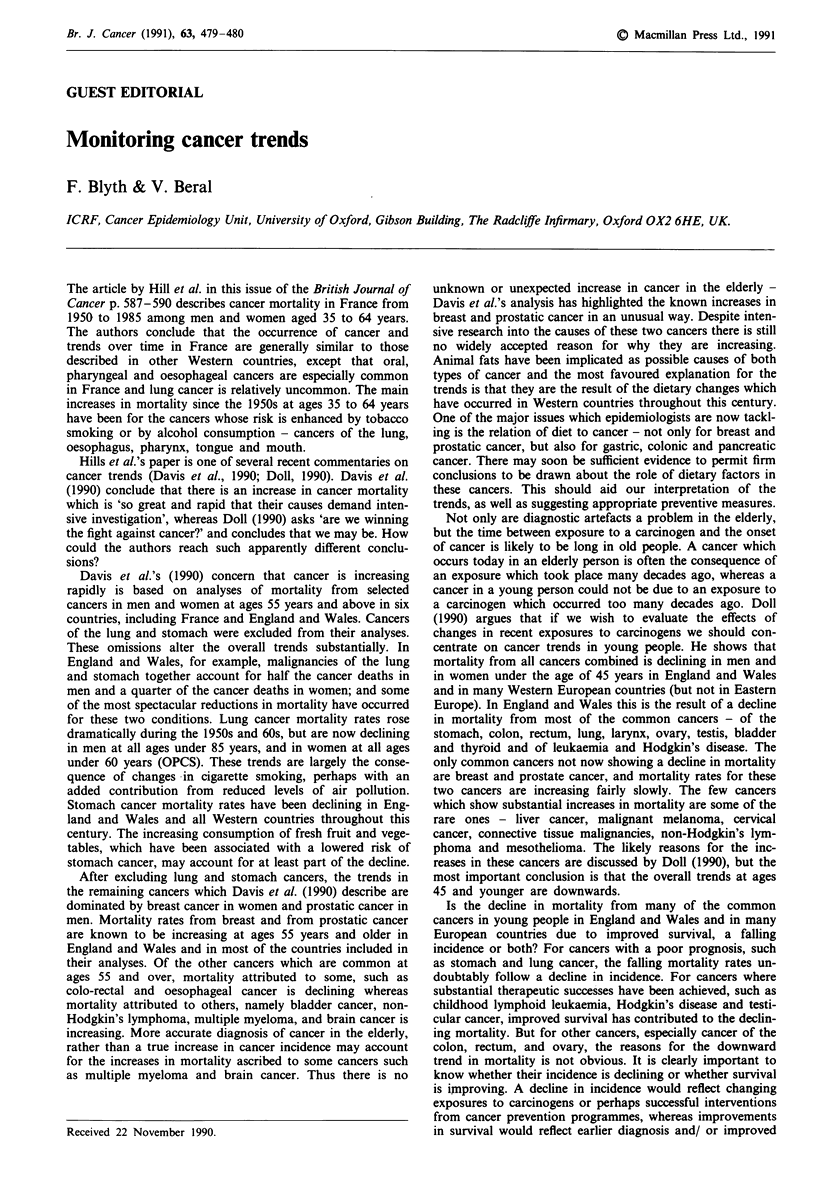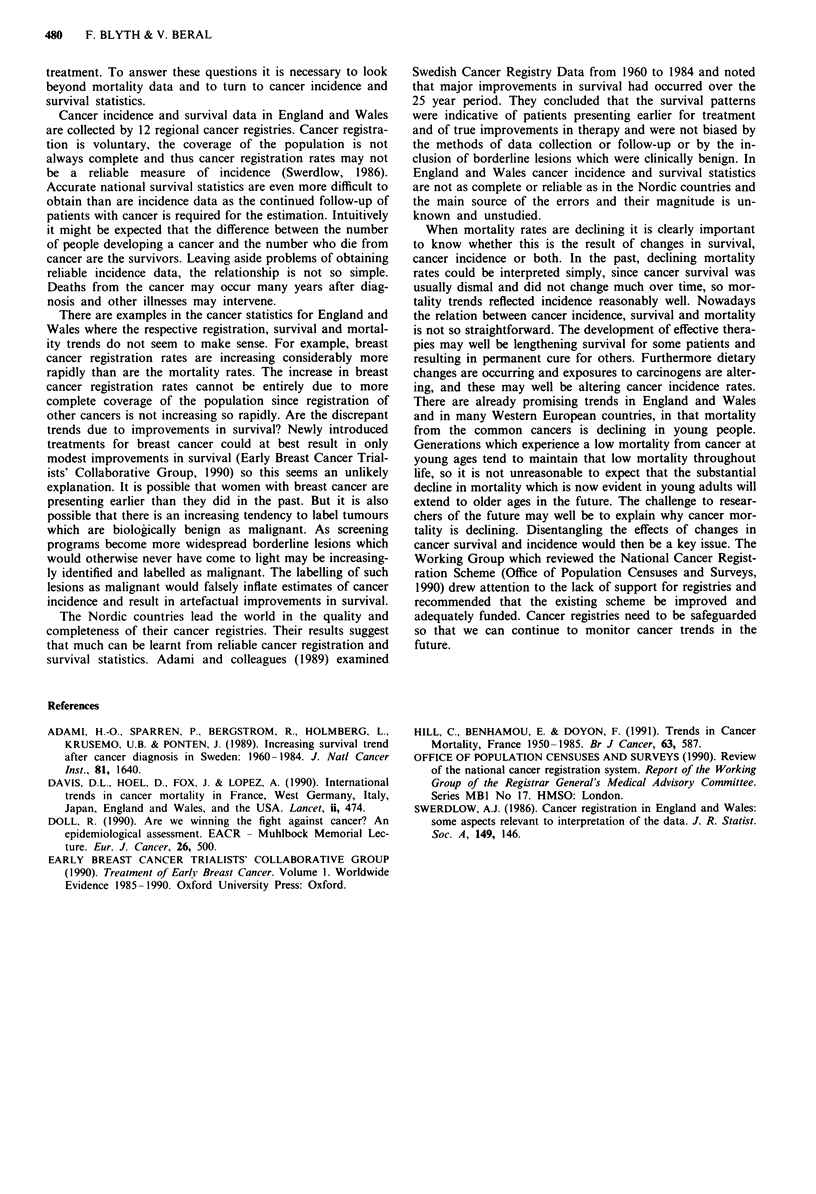# Monitoring cancer trends.

**DOI:** 10.1038/bjc.1991.113

**Published:** 1991-04

**Authors:** F. Blyth, V. Beral


Br. J. Cancer (1991), 63, 479–480 © Macmillan Press Ltd., 1991

GUEST EDITORIAL

### Monitoring cancer trends

F. Blyth & V. Beral

*ICRF, Cancer Epidemiology Unit, University of Oxford, Gibson Building, The Radcliffe Infirmary, Oxford OX2 6HE, UK.*

The article by Hill *et al.* in this issue of the *British Journal of Cancer* p. 587–590 describes cancer mortality in France from 1950 to 1985 among men and women aged 35 to 64 years. The authors conclude that the occurrence of cancer and trends over time in France are generally similar to those described in other Western countries, except that oral, pharyngeal and oesophageal cancers are especially common in France and lung cancer is relatively uncommon. The main increases in mortality since the 1950s at ages 35 to 64 years have been for the cancers whose risk is enhanced by tobacco smoking or by alcohol consumption – cancers of the lung, oesophagus, pharynx, tongue and mouth.

Hills *et al.*'s paper is one of several recent commentaries on cancer trends (Davis *et al.*, 1990; Doll, 1990). Davis *et al.* (1990) conclude that there is an increase in cancer mortality which is 'so great and rapid that their causes demand intensive investigation', whereas Doll (1990) asks 'are we winning the fight against cancer?' and concludes that we may be. How could the authors reach such apparently different conclusions?

Davis *et al.*'s (1990) concern that cancer is increasing rapidly is based on analyses of mortality from selected cancers in men and women at ages 55 years and above in six countries, including France and England and Wales. Cancers of the lung and stomach were excluded from their analyses. These omissions alter the overall trends substantially. In England and Wales, for example, malignancies of the lung and stomach together account for half the cancer deaths in men and a quarter of the cancer deaths in women; and some of the most spectacular reductions in mortality have occurred for these two conditions. Lung cancer mortality rates rose dramatically during the 1950s and 60s, but are now declining in men at all ages under 85 years, and in women at all ages under 60 years (OPCS). These trends are largely the consequence of changes in cigarette smoking, perhaps with an added contribution from reduced levels of air pollution. Stomach cancer mortality rates have been declining in England and Wales and all Western countries throughout this century. The increasing consumption of fresh fruit and vegetables, which have been associated with a lowered risk of stomach cancer, may account for at least part of the decline.

After excluding lung and stomach cancers, the trends in the remaining cancers which Davis *et al.* (1990) describe are dominated by breast cancer in women and prostatic cancer in men. Mortality rates from breast and from prostatic cancer are known to be increasing at ages 55 years and older in England and Wales and in most of the countries included in their analyses. Of the other cancers which are common at ages 55 and over, mortality attributed to some, such as colo-rectal and oesophageal cancer is declining whereas mortality attributed to others, namely bladder cancer, non-Hodgkin's lymphoma, multiple myeloma, and brain cancer is increasing. More accurate diagnosis of cancer in the elderly, rather than a true increase in cancer incidence may account for the increases in mortality ascribed to some cancers such as multiple myeloma and brain cancer. Thus there is no unknown or unexpected increase in cancer in the elderly – Davis *et al.*'s analysis has highlighted the known increases in breast and prostatic cancer in an unusual way. Despite intensive research into the causes of these two cancers there is still no widely accepted reason for why they are increasing. Animal fats have been implicated as possible causes of both types of cancer and the most favoured explanation for the trends is that they are the result of the dietary changes which have occurred in Western countries throughout this century. One of the major issues which epidemiologists are now tackling is the relation of diet to cancer – not only for breast and prostatic cancer, but also for gastric, colonic and pancreatic cancer. There may soon be sufficient evidence to permit firm conclusions to be drawn about the role of dietary factors in these cancers. This should aid our interpretation of the trends, as well as suggesting appropriate preventive measures.

Not only are diagnostic artefacts a problem in the elderly, but the time between exposure to a carcinogen and the onset of cancer is likely to be long in old people. A cancer which occurs today in an elderly person is often the consequence of an exposure which took place many decades ago, whereas a cancer in a young person could not be due to an exposure to a carcinogen which occurred too many decades ago. Doll (1990) argues that if we wish to evaluate the effects of changes in recent exposures to carcinogens we should concentrate on cancer trends in young people. He shows that mortality from all cancers combined is declining in men and in women under the age of 45 years in England and Wales and in many Western European countries (but not in Eastern Europe). In England and Wales this is the result of a decline in mortality from most of the common cancers – of the stomach, colon, rectum, lung, larynx, ovary, testis, bladder and thyroid and of leukaemia and Hodgkin's disease. The only common cancers not now showing a decline in mortality are breast and prostate cancer, and mortality rates for these two cancers are increasing fairly slowly. The few cancers which show substantial increases in mortality are some of the rare ones – liver cancer, malignant melanoma, cervical cancer, connective tissue malignancies, non-Hodgkin's lymphoma and mesothelioma. The likely reasons for the increases in these cancers are discussed by Doll (1990), but the most important conclusion is that the overall trends at ages 45 and younger are downwards.

Is the decline in mortality from many of the common cancers in young people in England and Wales and in many European countries due to improved survival, a falling incidence or both? For cancers with a poor prognosis, such as stomach and lung cancer, the falling mortality rates undoubtably follow a decline in incidence. For cancers where substantial therapeutic successes have been achieved, such as childhood lymphoid leukaemia, Hodgkin's disease and testicular cancer, improved survival has contributed to the declining mortality. But for other cancers, especially cancer of the colon, rectum, and ovary, the reasons for the downward trend in mortality is not obvious. It is clearly important to know whether their incidence is declining or whether survival is improving. A decline in incidence would reflect changing exposures to carcinogens or perhaps successful interventions from cancer prevention programmes, whereas improvements in survival would reflect earlier diagnosis and/ or improved

Received 22 November 1990.

treatment. To answer these questions it is necessary to look beyond mortality data and to turn to cancer incidence and survival statistics.

Cancer incidence and survival data in England and Wales are collected by 12 regional cancer registries. Cancer registration is voluntary, the coverage of the population is not always complete and thus cancer registration rates may not be a reliable measure of incidence (Swerdlow, 1986). Accurate national survival statistics are even more difficult to obtain than are incidence data as the continued follow-up of patients with cancer is required for the estimation. Intuitively it might be expected that the difference between the number of people developing a cancer and the number who die from cancer are the survivors. Leaving aside problems of obtaining reliable incidence data, the relationship is not so simple. Deaths from the cancer may occur many years after diagnosis and other illnesses may intervene.

There are examples in the cancer statistics for England and Wales where the respective registration, survival and mortality trends do not seem to make sense. For example, breast cancer registration rates are increasing considerably more rapidly than are the mortality rates. The increase in breast cancer registration rates cannot be entirely due to more complete coverage of the population since registration of other cancers is not increasing so rapidly. Are the discrepant trends due to improvements in survival? Newly introduced treatments for breast cancer could at best result in only modest improvements in survival (Early Breast Cancer Trialists' Collaborative Group, 1990) so this seems an unlikely explanation. It is possible that women with breast cancer are presenting earlier than they did in the past. But it is also possible that there is an increasing tendency to label tumours which are biologically benign as malignant. As screening programs become more widespread borderline lesions which would otherwise never have come to light may be increasingly identified and labelled as malignant. The labelling of such lesions as malignant would falsely inflate estimates of cancer incidence and result in artefactual improvements in survival.

The Nordic countries lead the world in the quality and completeness of their cancer registries. Their results suggest that much can be learnt from reliable cancer registration and survival statistics. Adami and colleagues (1989) examined Swedish Cancer Registry Data from 1960 to 1984 and noted that major improvements in survival had occurred over the 25 year period. They concluded that the survival patterns were indicative of patients presenting earlier for treatment and of true improvements in therapy and were not biased by the methods of data collection or follow-up or by the inclusion of borderline lesions which were clinically benign. In England and Wales cancer incidence and survival statistics are not as complete or reliable as in the Nordic countries and the main source of the errors and their magnitude is unknown and unstudied.

When mortality rates are declining it is clearly important to know whether this is the result of changes in survival, cancer incidence or both. In the past, declining mortality rates could be interpreted simply, since cancer survival was usually dismal and did not change much over time, so mortality trends reflected incidence reasonably well. Nowadays the relation between cancer incidence, survival and mortality is not so straightforward. The development of effective therapies may well be lengthening survival for some patients and resulting in permanent cure for others. Furthermore dietary changes are occurring and exposures to carcinogens are altering, and these may well be altering cancer incidence rates. There are already promising trends in England and Wales and in many Western European countries, in that mortality from the common cancers is declining in young people. Generations which experience a low mortality from cancer at young ages tend to maintain that low mortality throughout life, so it is not unreasonable to expect that the substantial decline in mortality which is now evident in young adults will extend to older ages in the future. The challenge to researchers of the future may well be to explain why cancer mortality is declining. Disentangling the effects of changes in cancer survival and incidence would then be a key issue. The Working Group which reviewed the National Cancer Registration Scheme (Office of Population Censuses and Surveys, 1990) drew attention to the lack of support for registries and recommended that the existing scheme be improved and adequately funded. Cancer registries need to be safeguarded so that we can continue to monitor cancer trends in the future.

### References

ADAMI, H.-O., SPARREN, P., BERGSTROM, R., HOLMBERG, L., KRUSEMO, U.B. & PONTEN, J. (1989). Increasing survival trend after cancer diagnosis in Sweden: 1960–1984. *J. Natl Cancer Inst.*, **81**, 1640.

DAVIS, D.L., HOEL, D., FOX, J. & LOPEZ, A. (1990). International trends in cancer mortality in France, West Germany, Italy, Japan, England and Wales, and the USA. *Lancet*, **ii**, 474.

DOLL, R. (1990). Are we winning the fight against cancer? An epidemiological assessment. EACR – Muhlbock Memorial Lecture. *Eur. J. Cancer*, **26**, 500.

EARLY BREAST CANCER TRIALISTS' COLLABORATIVE GROUP (1990). *Treatment of Early Breast Cancer*. Volume 1. Worldwide Evidence 1985–1990. Oxford University Press: Oxford.

HILL, C., BENHAMOU, E. & DOYON, F. (1991). Trends in Cancer Mortality, France 1950–1985. *Br J Cancer*, **63**, 587.

OFFICE OF POPULATION CENSUSES AND SURVEYS (1990). Review of the national cancer registration system. *Report of the Working Group of the Registrar General's Medical Advisory Committee*. Series MB1 No 17. HMSO: London.

SWERDLOW, A.J. (1986). Cancer registration in England and Wales: some aspects relevant to interpretation of the data. *J. R. Statist. Soc. A*, **149**, 146.